# Supplementary material for: A Likelihood Approach for Real-Time Calibration of Stochastic Compartmental Epidemic Models
Source: PLoS Comput Biol. 2017 Jan 17;13(1):e1005257. doi: 10.1371/journal.pcbi.1005257 (PMC5240920; doi:10.1371/journal.pcbi.1005257)
Supplement: S1 File — (TAR.GZ) [file pcbi.1005257.s014.tar.gz › HSPH_Online-SI-Revision/EnKF07/n10-mild/n10-mild_EnKF_table4.pdf]

1	R0	{1.25046, 6.80278}	{2.72447, 7.21696}	{{1.62218, 0.0845846}, {3., 14.9567}}	{117.877, 108.605}	inf	141.	770.736	{338.644, 1237.98}	454.575	0.67408	newly	135.	999.15	{140., 1236.}	648.126	0.620441	Reff	0.976984	0.507618	{0.109393, 0.776505}	57.1863	ar	3516.	17 263.2	{15 867., 19 324.}	390.989
2	R0	{1.32013, 4.45847}	{2.61115, 3.72075}	{{2.2024, 0.822019}, {3., 5.58672}}	{101.944, 46.9526}	inf	141.	1040.65	{116.56, 4570.45}	652.426	0.560963	newly	183.	1390.53	{106., 5648.}	675.929	0.587547	Reff	0.815972	0.643612	{0.115066, 1.76941}	60.0427	ar	4420.	18 680.3	{15 682., 26 928.}	322.631
3	R0	{1.31785, 5.36796}	{2.68489, 3.89661}	{{2.25996, 2.51007}, {3., 4.5562}}	{103.733, 27.4099}	inf	276.	647.079	{20.5713, 1449.81}	183.483	0.399536	newly	309.	836.89	{30., 1411.}	217.531	0.447802	Reff	0.782671	0.736747	{0.23674, 1.58947}	67.4676	ar	4942.	18 022.5	{15 305., 22 140.}	264.681
4	R0	{1.38801, 6.16031}	{2.34446, 5.08261}	{{1.18713, 0.686411}, {3., 6.51954}}	{77.3968, 44.3934}	inf	192.	1324.48	{449.235, 4956.34}	596.253	0.622029	newly	196.	1706.9	{317., 5509.}	779.429	0.651461	Reff	0.811292	0.623645	{0.23141, 1.19995}	54.9526	ar	4790.	18 259.7	{15 417., 26 819.}	281.205
5	R0	{1.27955, 11.57}	{2.50516, 3.87931}	{{1.23031, 2.25487}, {3., 6.5956}}	{97.8345, 66.471}	inf	157.	916.751	{175.315, 3366.53}	493.345	0.570939	newly	94.	1040.58	{140., 2431.}	1007.38	0.755865	Reff	1.02351	0.323892	{0.0650289, 0.677684}	69.2309	ar	3460.	18 433.7	{15 910., 25 200.}	432.766
6	R0	{1.22184, 4.34395}	{2.22825, 6.74694}	{{0.608484, 0.500211}, {3., 10.1179}}	{112.262, 117.166}	inf	121.	478.371	{29.7249, 1116.17}	335.181	0.452307	newly	169.	650.89	{18., 1202.}	350.231	0.57444	Reff	0.87777	0.539161	{0.103467, 1.00515}	54.9743	ar	3524.	17 490.7	{15 827., 20 413.}	396.331
7	R0	{1.23856, 4.59068}	{2.60452, 6.88665}	{{2.24601, 0.201377}, {3., 16.3267}}	{120.147, 150.695}	inf	125.	768.727	{49.7515, 1838.54}	541.014	0.516353	newly	186.	1587.15	{29., 6889.}	812.21	0.657246	Reff	0.844822	0.711132	{0.0657965, 1.73603}	64.7938	ar	3855.	18 246.9	{15 525., 24 576.}	373.33
8	R0	{1.34531, 10.6689}	{2.63733, 5.62006}	{{1.59909, 3.24965}, {3., 6.95679}}	{96.0386, 51.9833}	inf	364.	788.314	{194.595, 2476.42}	148.398	0.265576	newly	224.	903.91	{190., 3069.}	310.424	0.304962	Reff	0.92221	0.523812	{0.15003, 0.66906}	60.2716	ar	4852.	18 066.6	{16 372., 22 669.}	272.354
9	R0	{1.31775, 5.19226}	{2.67333, 5.30897}	{{1.92751, 3.52589}, {3., 7.25897}}	{102.871, 35.6249}	inf	255.	532.903	{65.4757, 906.644}	161.101	0.308764	newly	288.	551.3	{51., 1738.}	146.646	0.390019	Reff	0.778658	0.659769	{0.0744573, 1.44018}	55.1439	ar	4708.	17 502.2	{15 057., 24 109.}	271.754
10	R0	{1.25916, 6.89207}	{2.78115, 3.89541}	{{2.29176, 0.354615}, {3., 5.28915}}	{120.873, 43.4798}	inf	145.	639.688	{493.469, 848.188}	341.362	0.581461	newly	135.	1183.58	{420., 2719.}	776.726	0.758367	Reff	0.888841	0.707054	{0.227236, 1.19947}	49.9357	ar	3823.	18 059.1	{16 520., 21 158.}	372.38
11	R0	{1.20377, 5.46566}	{2.35665, 7.09691}	{{0.961584, 3.80862}, {3., 14.6209}}	{106.608, 71.6833}	inf	117.	257.49	{86.665, 177.41}	128.393	0.15158	newly	147.	164.99	{57., 165.}	58.0612	0.18517	Reff	0.911254	0.386313	{0.0943978, 0.76544}	68.8735	ar	3114.	16 786.9	{15 751., 19 132.}	439.078
12	R0	{1.27878, 2.55804}	{2.66621, 11.2981}	{{2.16846, 6.51446}, {3., 15.7832}}	{108.496, 341.672}	inf	51.	361.954	{87.6679, 282.82}	621.605	0.559432	newly	100.	209.2	{39., 137.}	152.5	0.302942	Reff	0.804992	0.585712	{0.214126, 1.02229}	60.7813	ar	3930.	16 717.4	{15 224., 18 978.}	325.38
13	R0	{1.41227, 5.45696}	{2.63177, 5.12405}	{{1.52571, 3.23516}, {3., 6.97583}}	{86.3503, 35.0477}	inf	198.	895.03	{237.521, 2685.71}	361.639	0.463799	newly	224.	1090.66	{240., 3738.}	393.17	0.396195	Reff	0.81262	0.501221	{0.140581, 0.678458}	59.9341	ar	4822.	18 183.	{16 318., 22 434.}	277.083
14	R0	{1.32917, 7.27593}	{2.81114, 4.66555}	{{2.38553, 2.09733}, {3., 14.5661}}	{111.496, 57.9443}	inf	261.	705.127	{258.351, 1998.41}	177.165	0.268351	newly	225.	690.57	{253., 1846.}	212.698	0.337412	Reff	0.839903	0.427568	{0.0440145, 0.943703}	61.5771	ar	4758.	18 359.7	{16 239., 23 621.}	285.87
15	R0	{1.31629, 9.97481}	{2.51982, 6.40314}	{{1.31339, 1.05572}, {3., 9.35591}}	{91.5946, 35.8069}	inf	321.	1428.53	{425.1, 4168.88}	348.195	0.377661	newly	226.	1633.	{233., 4023.}	624.558	0.520339	Reff	0.982216	0.648585	{0.229747, 1.33865}	59.3779	ar	4543.	19 468.5	{16 089., 28 433.}	328.539
16	R0	{1.36735, 5.74166}	{2.70077, 8.289}	{{1.98018, 3.53638}, {3., 14.82}}	{97.5189, 79.7017}	inf	216.	639.773	{57.004, 995.978}	230.103	0.44402	newly	224.	776.98	{42., 1952.}	289.482	0.498888	Reff	0.874557	0.89253	{0.0505721, 1.97985}	50.3818	ar	4516.	18 156.	{15 488., 24 994.}	302.038
17	R0	{1.27524, 13.2396}	{2.72704, 8.41806}	{{2.24824, 3.80438}, {3., 18.443}}	{113.845, 48.3174}	inf	156.	1190.94	{98.7603, 760.499}	676.505	0.726552	newly	82.	943.98	{138., 1028.}	1064.07	0.865132	Reff	1.15027	0.50186	{0.0911634, 1.2786}	73.3595	ar	3731.	19 237.2	{16 963., 25 331.}	415.604
18	R0	{1.21884, 7.71545}	{2.71856, 3.63027}	{{2.20919, 3.32273}, {3., 4.23158}}	{123.045, 52.948}	inf	198.	1096.36	{188.66, 3155.53}	458.267	0.530913	newly	194.	1897.09	{236., 6461.}	879.479	0.701593	Reff	0.931559	0.866843	{0.40711, 2.20897}	52.5797	ar	3080.	19 436.5	{15 135., 30 335.}	531.055
19	R0	{1.34879, 6.71464}	{2.49991, 5.04718}	{{1.72599, 3.36688}, {3., 6.21485}}	{85.3448, 24.8331}	inf	164.	253.659	{41.9954, 542.82}	161.194	0.486534	newly	162.	387.18	{41., 579.}	248.728	0.546452	Reff	0.798079	0.488741	{0.207457, 0.8292}	54.707	ar	4585.	16 080.3	{14 923., 18 877.}	250.715
20	R0	{1.37284, 5.75036}	{2.75375, 3.86469}	{{2.20564, 3.84997}, {3., 3.89475}}	{100.588, 32.7922}	inf	225.	586.299	{93.7775, 1461.32}	183.923	0.32961	newly	244.	1343.49	{151., 4543.}	467.357	0.455207	Reff	0.87848	0.984689	{0.197563, 2.20542}	72.533	ar	4750.	18 218.9	{15 488., 25 946.}	283.555
21	R0	{1.29938, 5.36419}	{2.73046, 3.64809}	{{2.20635, 0.699}, {3., 5.79437}}	{113.405, 40.8901}	inf	178.	687.306	{35.7796, 1039.84}	309.525	0.436149	newly	209.	865.22	{82., 2079.}	333.282	0.452399	Reff	0.808604	0.6122	{0.0553661, 2.15905}	69.8382	ar	4337.	17 653.9	{15 369., 24 318.}	307.053
22	R0	{1.31882, 7.47626}	{2.77854, 9.24493}	{{2.18684, 0.0304083}, {3., 18.6738}}	{110.684, 79.1002}	inf	191.	595.107	{177.341, 1870.55}	219.502	0.367583	newly	169.	587.7	{99., 920.}	268.84	0.428076	Reff	0.929504	0.611846	{0.114752, 1.50753}	60.6173	ar	3977.	18 257.3	{16 758., 21 046.}	359.073
23	R0	{1.26215, 3.46375}	{2.20465, 3.41773}	{{0.948735, 2.17099}, {3., 4.52505}}	{93.3069, 33.7547}	inf	114.	329.17	{172.667, 316.478}	195.673	0.287915	newly	207.	472.3	{143., 409.}	150.377	0.204981	Reff	0.848291	0.489059	{0.14694, 0.762138}	56.6852	ar	3607.	16 653.8	{15 553., 17 049.}	361.707
24	R0	{1.16712, 4.19464}	{2.45096, 4.05107}	{{0.0676989, 3.54436}, {3., 6.41106}}	{138.831, 20.7406}	inf	82.	382.582	{98.3477, 158.499}	377.375	0.408685	newly	105.	378.69	{112., 785.}	271.781	0.387115	Reff	0.873473	0.509676	{0.0115329, 0.939471}	54.7836	ar	3000.	16 813.3	{15 629., 20 159.}	460.445
25	R0	{1.22751, 3.34517}	{2.34321, 7.09678}	{{0.632544, 0.360988}, {3., 11.2327}}	{115.082, 179.72}	inf	114.	517.581	{28.9166, 627.989}	390.982	0.447266	newly	241.	737.39	{20., 1331.}	286.17	0.63195	Reff	0.932662	0.579317	{0.109587, 1.09788}	57.3102	ar	3086.	17 550.4	{15 921., 21 620.}	468.71
26	R0	{1.18747, 5.88083}	{2.65165, 7.87669}	{{1.74198, 3.24214}, {3., 19.2427}}	{123.302, 77.013}	inf	107.	918.1	{535.619, 688.967}	761.928	0.813222	newly	102.	1080.07	{186., 1453.}	962.814	0.753539	Reff	0.802017	0.528366	{0.239337, 0.846878}	56.4521	ar	3614.	16 978.3	{15 473., 21 808.}	369.791
27	R0	{1.29023, 4.76202}	{2.78796, 5.29289}	{{2.44989, 1.52034}, {3., 16.7544}}	{116.082, 57.3257}	inf	148.	965.639	{366.496, 3576.31}	558.029	0.540302	newly	192.	1224.28	{268., 3306.}	547.281	0.529357	Reff	0.787814	0.661152	{0.0789032, 1.4395}	56.1409	ar	4245.	17 662.8	{15 156., 24 355.}	316.085
28	R0	{1.2258, 12.9056}	{2.42392, 4.81966}	{{0.938774, 1.49026}, {3., 5.70635}}	{110.681, 62.6545}	inf	171.	234.71	{5.58074, 437.894}	137.54	0.731506	newly	91.	224.11	{5., 384.}	231.33	0.714345	Reff	0.929156	0.29625	{0.064894, 0.621465}	68.1162	ar	3639.	12 006.4	{11 164., 13 557.}	229.937
29	R0	{1.34702, 6.29049}	{2.33926, 6.04507}	{{1.18883, 0.875876}, {3., 9.99303}}	{80.6684, 69.9648}	inf	220.	767.633	{312.737, 713.366}	260.323	0.419615	newly	234.	940.52	{220., 1020.}	311.838	0.383644	Reff	0.838924	0.537584	{0.163052, 1.19037}	56.5646	ar	4560.	17 200.9	{15 340., 22 075.}	277.214
30	R0	{1.37931, 6.78741}	{2.73969, 4.51074}	{{2.19211, 0.527232}, {3., 16.9873}}	{102.608, 76.5245}	inf	216.	827.036	{383.7, 1701.35}	284.975	0.441485	newly	206.	1129.41	{161., 2164.}	457.053	0.561439	Reff	0.813793	0.560454	{0.346331, 0.848754}	45.8221	ar	4667.	17 856.2	{15 336., 22 793.}	282.605
31	R0	{1.16062, 4.47529}	{2.27287, 6.76535}	{{0.62064, 0.429147}, {3., 10.4928}}	{121.594, 117.985}	inf	144.	472.005	{33.4212, 1111.3}	282.571	0.521785	newly	207.	698.71	{23., 1045.}	308.671	0.643965	Reff	0.868956	0.583605	{0.114646, 1.04275}	55.026	ar	3037.	17 527.8	{15 840., 19 881.}	477.142
32	R0	{1.37647, 18.7122}	{2.41488, 3.73005}	{{0.830905, 1.8699}, {3., 7.23065}}	{96.7649, 80.0662}	inf	353.	407.315	{102.341, 308.655}	55.5704	0.210353	newly	116.	468.85	{73., 609.}	319.56	0.574793	Reff	0.957197	0.403643	{0.150395, 0.667459}	58.257	ar	4824.	15 456.8	{14 687., 15 434.}	220.415
33	R0	{1.35084, 6.08841}	{2.33271, 5.87626}	{{1.14628, 1.05056}, {3., 9.41484}}	{81.3828, 65.9185}	inf	251.	1142.43	{389.002, 3777.9}	359.232	0.393865	newly	266.	1523.25	{208., 3275.}	487.38	0.420158	Reff	0.778759	0.589626	{0.174417, 1.15242}	58.8689	ar	4759.	18 285.6	{15 595., 26 787.}	284.232
34	R0	{1.36912, 6.316}	{2.51112, 5.8651}	{{1.22362, 1.15274}, {3., 9.02752}}	{88.1746, 58.1817}	inf	243.	1042.22	{415.169, 4000.36}	334.661	0.419468	newly	247.	1345.86	{230., 3225.}	450.753	0.417823	Reff	0.921965	0.599251	{0.185258, 1.22147}	57.4045	ar	4537.	17 892.3	{15 454., 26 684.}	294.364
35	R0	{1.35831, 7.09038}	{2.44406, 6.67836}	{{1.16172, 0.645063}, {3., 10.7575}}	{86.7968, 66.7673}	inf	210.	1121.17	{497.662, 3310.21}	442.199	0.564496	newly	184.	1313.93	{239., 2081.}	618.332	0.565018	Reff	0.809553	0.582803	{0.102186, 1.18669}	62.138	ar	4667.	18 227.6	{15 702., 25 571.}	290.564
36	R0	{1.28315, 5.89938}	{2.71279, 5.23627}	{{2.20688, 0.213894}, {3., 16.3501}}	{116.013, 120.859}	inf	195.	686.636	{63.9052, 1268.4}	281.911	0.441789	newly	206.	1409.02	{28., 5030.}	629.388	0.743147	Reff	0.765399	0.591972	{0.20573, 1.19142}	48.1537	ar	4460.	17 305.5	{15 453., 23 024.}	288.017
37	R0	{1.19303, 2.75113}	{2.72032, 11.5351}	{{2.27884, 6.61949}, {3., 15.2762}}	{128.018, 319.286}	inf	59.	473.488	{135.187, 237.778}	706.635	0.61638	newly	124.	285.39	{59., 217.}	166.008	0.284461	Reff	0.868407	0.756432	{0.360259, 1.40242}	52.2726	ar	3142.	17 268.9	{15 491., 18 420.}	449.616
38	R0	{1.36033, 7.8088}	{2.65386, 2.88337}	{{1.95914, 2.11498}, {3., 3.54169}}	{95.0897, 63.0753}	inf	289.	561.922	{40.6317, 948.384}	151.846	0.389088	newly	222.	1592.52	{62., 6185.}	652.441	0.566976	Reff	0.859593	0.861364	{0.268879, 1.96191}	65.3671	ar	4941.	18 096.2	{15 319., 24 880.}	266.247
39	R0	{1.27353, 3.70845}	{2.57552, 6.67}	{{2.23099, 0.220222}, {3., 16.4269}}	{111.567, 182.241}	inf	54.	512.236	{47.7352, 596.317}	859.199	0.741823	newly	83.	1485.08	{25., 4500.}	1717.16	0.825094	Reff	0.815441	0.768985	{0.138282, 1.6277}	62.3688	ar	3725.	17 632.8	{15 532., 24 331.}	373.362
40	R0	{1.19983, 12.1486}	{2.62186, 8.85065}	{{1.69488, 0.705622}, {3., 13.8688}}	{118.519, 42.3745}	inf	143.	1571.61	{647.77, 4824.35}	1002.33	0.802182	newly	81.	1610.2	{286., 5346.}	1891.33	1.02359	Reff	0.938027	1.05925	{0.433223, 2.36974}	56.3144	ar	3619.	19 672.7	{16 335., 30 874.}	443.595
41	R0	{1.20935, 2.67169}	{2.32431, 7.00169}	{{0.561974, 1.50535}, {3., 9.52779}}	{116.859, 185.589}	inf	33.	389.886	{20.258, 1052.24}	1098.08	0.870868	newly	73.	504.24	{11., 1558.}	624.658	0.689015	Reff	0.803008	0.637513	{0.0976151, 1.4024}	63.4821	ar	3429.	17 518.4	{15 648., 21 005.}	410.891
42	R0	{1.23313, 4.74781}	{2.75865, 5.28473}	{{2.1955, 0.115822}, {3., 6.29711}}	{123.711, 77.4824}	inf	154.	844.414	{90.4402, 682.913}	466.032	0.582779	newly	207.	1099.37	{69., 2843.}	458.111	0.572952	Reff	0.853203	0.535062	{0.203651, 0.919798}	46.905	ar	3835.	17 272.7	{15 041., 19 595.}	350.395
43	R0	{1.26105, 2.39713}	{2.51085, 2.2101}	{{1.62869, 0.593481}, {3., 3.31525}}	{99.108, 52.9972}	inf	51.	196.076	{31.6306, 359.987}	298.496	0.519544	newly	120.	473.1	{58., 732.}	318.067	0.455611	Reff	0.808711	0.505942	{0.200409, 0.883268}	53.3626	ar	3723.	16 072.6	{15 144., 16 438.}	331.71
44	R0	{1.36948, 8.88006}	{2.50935, 5.32053}	{{1.25099, 1.62932}, {3., 7.22819}}	{86.9652, 40.0845}	inf	313.	812.131	{262.683, 2629.37}	194.109	0.267925	newly	232.	1407.26	{169., 5734.}	531.698	0.370192	Reff	0.929192	0.701094	{0.226637, 1.627}	57.6747	ar	4437.	17 281.6	{14 959., 23 974.}	289.487
45	R0	{1.34882, 3.3791}	{2.31822, 6.42476}	{{1.41826, 3.49646}, {3., 8.45968}}	{71.8704, 90.1322}	inf	128.	514.792	{197.756, 589.648}	302.468	0.39243	newly	208.	586.7	{156., 595.}	201.933	0.206124	Reff	0.796074	0.438755	{0.0967221, 0.989638}	60.1437	ar	4422.	18 587.6	{16 796., 24 915.}	320.344
46	R0	{1.23774, 4.34917}	{2.5363, 11.8682}	{{1.78958, 1.52152}, {3., 17.2713}}	{104.914, 217.01}	inf	149.	391.828	{37.0439, 430.414}	202.545	0.36538	newly	217.	339.44	{19., 591.}	147.143	0.538664	Reff	0.844634	0.610849	{0.0731268, 1.84584}	68.0119	ar	3685.	17 069.3	{15 381., 20 868.}	363.211
47	R0	{1.34327, 4.78811}	{2.59779, 11.1601}	{{1.77907, 0.973313}, {3., 19.9076}}	{93.3931, 206.268}	inf	220.	328.619	{79.7669, 461.982}	88.308	0.305042	newly	304.	455.74	{32., 942.}	139.441	0.562933	Reff	0.872454	0.625131	{0.0946109, 1.52255}	61.1775	ar	4517.	16 607.9	{15 275., 20 023.}	267.675
48	R0	{1.24366, 4.25814}	{2.63918, 5.35661}	{{1.97761, 3.53426}, {3., 7.49042}}	{112.21, 41.4369}	inf	141.	236.902	{104.946, 432.723}	94.2631	0.219264	newly	200.	406.88	{78., 963.}	167.95	0.37554	Reff	0.862976	0.639381	{0.139679, 1.18627}	59.2798	ar	3678.	16 431.1	{14 889., 19 663.}	346.74
49	R0	{1.36017, 7.5415}	{2.61906, 7.69744}	{{1.58903, 0.0127699}, {3., 14.046}}	{92.5538, 68.2661}	inf	178.	395.299	{102.439, 262.861}	141.745	0.278852	newly	164.	295.59	{54., 487.}	119.128	0.367194	Reff	1.01999	0.600675	{0.287848, 0.960485}	46.1047	ar	4178.	16 403.3	{15 545., 18 315.}	292.61
50	R0	{1.25333, 6.63578}	{2.65161, 11.5262}	{{1.63235, 5.51336}, {3., 15.5215}}	{111.565, 77.6464}	inf	109.	352.848	{123.711, 485.463}	232.197	0.418678	newly	93.	237.55	{52., 256.}	169.774	0.2751	Reff	0.878334	0.642829	{0.187189, 1.63118}	57.7351	ar	3401.	15 664.4	{14 225., 21 354.}	360.581
